# Viral Etiology of Chronic Obstructive Pulmonary Disease Exacerbations during the A/H1N1pdm09 Pandemic and Postpandemic Period

**DOI:** 10.1155/2015/560679

**Published:** 2015-05-07

**Authors:** Ivan Sanz, Sonia Tamames, Silvia Rojo, Mar Justel, José Eugenio Lozano, Carlos Disdier, Tomás Vega, Raúl Ortiz de Lejarazu

**Affiliations:** ^1^Valladolid National Influenza Centre, Avenida Ramón y Cajal No. 7, 47005 Valladolid, Spain; ^2^Microbiology and Immunology Service, University Clinic Hospital of Valladolid, Avenida Ramón y Cajal s/n, 47005 Valladolid, Spain; ^3^Consejería de Sanidad, Junta de Castilla y León, Paseo de Zorrilla No. 1, 47007 Valladolid, Spain; ^4^Pulmonology Service, University Clinic Hospital of Valladolid, Avenida Ramón y Cajal s/n, 47005 Valladolid, Spain

## Abstract

Viral infections are one of the main causes of acute exacerbations of chronic obstructive pulmonary disease (AE-COPD). Emergence of A/H1N1pdm influenza virus in the 2009 pandemic changed the viral etiology of exacerbations that were reported before the pandemic. The aim of this study was to describe the etiology of respiratory viruses in 195 Spanish patients affected by AE-COPD from the pandemic until the 2011-12 influenza epidemic. During the study period (2009–2012), respiratory viruses were identified in 48.7% of samples, and the proportion of viral detections in AE-COPD was higher in patients aged 30–64 years than ≥65 years. Influenza A viruses were the pathogens most often detected during the pandemic and the following two influenza epidemics in contradistinction to human rhino/enteroviruses that were the main viruses causing AE-COPD before the pandemic. The probability of influenza virus detection was 2.78-fold higher in patients who are 30–64 years old than those ≥65. Most respiratory samples were obtained during the pandemic, but the influenza detection rate was higher during the 2011-12 epidemic. There is a need for more accurate AE-COPD diagnosis, emphasizing the role of respiratory viruses. Furthermore, diagnosis requires increased attention to patient age and the characteristics of each influenza epidemic.

## 1. Introduction

Chronic obstructive pulmonary disease (COPD) is a slowly progressive and largely irreversible clinical condition characterized by airflow limitation [1]. In Spain, COPD affects over 10% of the population between 40 and 80 years of age [2, 3]. Acute exacerbations of COPD (AE-COPD) play a crucial role in the course of the disease, having a negative impact on morbidity, mortality, healthcare costs, and health-related quality of life [4, 5]. Patients with moderate and severe COPD are prone to exacerbations, and the frequency of these episodes increases with the severity of disease [6]. One of the key points in COPD management programs is prevention and treatment of exacerbations [7]. Results of follow-up studies show that patients who suffer a high number of exacerbations during a given period of time will continue to suffer frequent exacerbations in the future [8]. Therefore, the frequency of exacerbations will depend on the patient's underlying severity of lung disease and number of prior exacerbations [9].

The etiology of AE-COPD is diverse. Most AE-COPD cases are attributed to bacterial or viral respiratory infections [10] and to both types of microorganisms together [11]. However to a minor extent, exacerbations are also associated with pollution, tobacco consumption, temperature changes, allergens, and other comorbidities such as heart failure and pulmonary thromboembolism [8, 12]. Respiratory viral infections have been associated with more frequent and severe AE-COPD and also with longer recovery times than episodes caused by other factors including bacteria [13, 14]. Studies conducted before emergence of the pandemic H1N1pdm09 strain showed that half of all AE-COPD cases were associated with viral infections and that picornaviruses (especially human rhinovirus and enterovirus (HREV)) were the dominant viral pathogens diagnosed in these patients [15, 16]. HREVs are the main viruses responsible for the common cold, with high prevalence throughout the whole year and without an established epidemic circulation period. Currently they are the most important trigger of COPD exacerbations [17]. Related with that, exacerbations treated with antibiotics could lead to the emergence of resistances in cases with other etiologies, which constitutes a problem particularly in southern Mediterranean European countries where antibiotics are widely used for these kinds of patients [18]. Improvement of clinical diagnosis and correct identification of respiratory viruses may help reduce the use of these antibiotics. It is important to find clinical and analytical parameters to guide identification of the etiology of new AE-COPD cases, especially considering the laborious techniques currently used for diagnosis [19].

In 2009 the world experienced the first pandemic of influenza A virus in 40 years, and this pathogen is now known as the H1N1pdm09 pandemic virus [20]. During 2009-10, this virus spread worldwide, causing high infection rates but low mortality compared with previous pandemics (Spanish Flu, Asian Flu, and Hong Kong Flu) [21]. The pandemic resulted in a high rate of screening for respiratory viruses in patients with respiratory clinical manifestations, including those with COPD. During this pandemic and following influenza epidemics, Valladolid National Influenza Centre (Valladolid NIC, Spain) and the Microbiology & Immunology Service of Clinic University Hospital of Valladolid worked closely on several topics related to influenza A/H1N1pdm09 [22, 23]. Consequently, we received a large number of respiratory samples from the Hospital Network of Castile and León (2.5 million habitants) and currently we serve as a reference center for viral diagnostics for influenza-suspect cases and for other respiratory pathologies, including AE-COPD.

The aim of this study is to describe the etiological characteristics of respiratory viruses linked to COPD exacerbations after a singular pandemic period caused by a new influenza virus. We have placed special emphasis on the differences of viral etiology of AE-COPD between the pandemic and the following postpandemic period.

## 2. Materials and Methods

### 2.1. Study Design

This retrospective observational study was done at the Microbiology & Immunology Service of the Clinic University Hospital of Valladolid, Valladolid, Spain. Respiratory samples from 195 AE-COPD patients hospitalized in Castile and León Hospital Network (Spain) were sent to the microbiology laboratory for viral molecular diagnosis between October 2009 and September 2012. This work was exempt from Ethical Committee approval and from the need for informed consent following Spanish laws regarding the use of routine clinical samples in research studies.

### 2.2. Case Definition

Sample recruitment was done reviewing microbiology laboratory order slips. Only cases in which the microbiology order slip specifically showed COPD exacerbation causing respiratory sample submission were included. This inclusion criterion was established by the chief pulmonologist of our hospital network following the Anthonisen criteria [24] in which one of the following symptoms were present: cough, dyspnea, or sputum increasing in volume or purulence. Before the sample was included in the study, the clinical chart of each of the potentially included patients was checked for the presence of at least one of the cited symptoms. Demographic data such as age and sex were also obtained from the order slips. Clinical samples from AE-COPD patients without Anthonisen symptoms or who lacked clinical or demographic information were excluded from this study. Because COPD is a disease that affects only adults [3], only patients ≥30 years old were included in this work.

### 2.3. Epidemic Information

The infection rates and prevalence of the different respiratory viruses diagnosed in this work were obtained from local epidemiological surveillance data. This free information is provided weekly by the public health authorities through the Influenza Sentinel Surveillance Network (ISSN) of Castile and León. We defined six study periods (Table 1) following the World Health Organization guidelines [25].

### 2.4. Viral Analysis

Viral detection was done by means of a set of molecular diagnostic techniques implemented in the lab routine during the 2009 pandemic. Briefly, genetic material was extracted from the respiratory samples by using an* EasyMag* (*Biomerieux*,* Craponne*,* France*) automatic extractor, and the eluted final volume of 50 *μ*L was used for multiple molecular diagnostic assays. Primary screening was by multiplex real-time polymerase chain reaction (RT-PCR) for 17 different respiratory viral targets (influenza virus A/H3, A/H1, A/H1N1pdm09 and B; respiratory syncytial viruses A and B (RSV A and RSV B); HREV; coronavirus OC43, 229E, HKU1, and NL63; metapneumovirus; parainfluenza 1, 2, 3, and 4; adenovirus; and bocavirus) using* Luminex 200* platform (Luminex, Austin, TX, USA) and* Respiratory Viral Panel-XTAG RVP* (Abbott, Chicago, IL, USA). Influenza A viruses not subtyped by this technique were identified by means of real-time RT-PCR* Roche 2.0* platform (Roche, Basel, Switzerland) using* Influenza A/H1N1 Detection Set* (Roche) for the specific detection of A/H1N1pdm09 virus. Also, to specifically characterize 16 haemagglutinin and 9 neuraminidase types of non subtypable influenza A viruses, we used* Clondiag Array Mate* and* Influenza A Genotyping* reagents (Alere, Waltham, MA, USA). Influenza B lineages Victoria and Yamagata were identified using a real-time RT-PCR as previously described [26].

### 2.5. Data Analysis

This study included data from two groups of patients: those who were 30 to 64 years old and those ≥65 years. A descriptive analysis was conducted by calculating the appropriate summary measures for quantitative and qualitative variables. Means and standard deviations were calculated for continuous variables. Associations from basic clinical data and frequency of viral infections were analyzed by Student's *t*-test adjusted by age with 95% confidence interval (*α* = 0.01). Detection probability of the different respiratory pathogens involved in this work was calculated using odds ratio (OR) adjusted by different demographic and epidemiological characteristics such as sex, age, and the influenza circulation periods included in the study. OR was analyzed using 95% confidence interval (CI95%) and *α* = 0.05%. The statistical package employed was SPSS 19.0.

## 3. Results

Of the 195 patients included in the study, 94 (48.2%) were between the ages of 30 and 64 years and 101 (51.8%) were ≥65 years. The average age of all patients was 63.9 ± 13.1 years old and 136 were males (69.7%). From September 2009 until September 2012, respiratory viruses were diagnoses in 95 AE-COPD samples (48.7%), and no pathogen was detected in 100 samples (51.3%). The most frequently detected respiratory virus during this period was influenza A/H1N1pdm09, present in 41 cases (21.0%), followed by HREV (*n* = 24; 12.3%), RSV (*n* = 13; 6.7%), and H3N2 influenza virus (*n* = 11; 5.6%). Twelve samples (6.2%) were positive for other respiratory pathogens included in the molecular diagnostic assays. Influenza B virus was detected in only one AE-COPD patient (0.5%), and 5 cases (2.6%) of coinfection were also detected.

**Figure 1:**
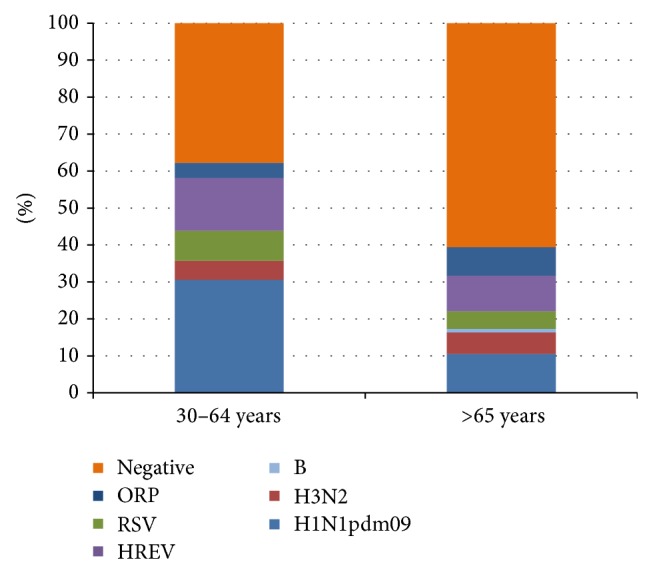
Cumulative percentage of respiratory virus prevalence causing AE-COPD in adults aged 30–64 years and elderly patients aged ≥65 years. The presence of viruses in AE-COPD declined with the age of individuals in the study. ORP: other respiratory pathogens; HREV: human rhino-enterovirus.

Viral diagnostic findings in AE-COPD patients decreased with age. Viruses were found in 61 samples (62.2%) from patients in the 30–64-year age group and in 41 patients (38.0%) in the ≥65-year age group (Figure 1). There was no age difference between AE-COPD patients with viral infection (62.1 ± 1.5 years) and those that tested negative by molecular diagnostics (65.6 ± 1.4 years, *p* = 0.11). Influenza A/H1N1pdm09 was the most often detected virus in the 30–64-year age group (*n* = 30; 30.6%). On the other hand, most of the viruses had prevalences similar to one another in the ≥65-year age group: A/H1N1pdm09, 10.2%; HREV, 9.3%; other respiratory pathogens, 7.4%. Thus the absence of positive diagnostics was more common in this age group.

**Figure 2:**
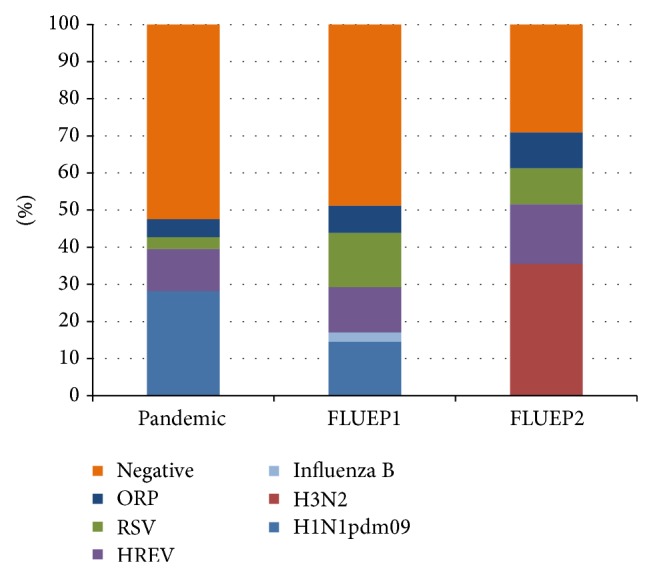
Cumulative percentage distribution of respiratory viral pathogens causing AE-COPD episodes during the 2009 pandemic and influenza epidemics described in the study. ORP: other respiratory pathogens; HREV: human rhino-enterovirus.

**Figure 3:**
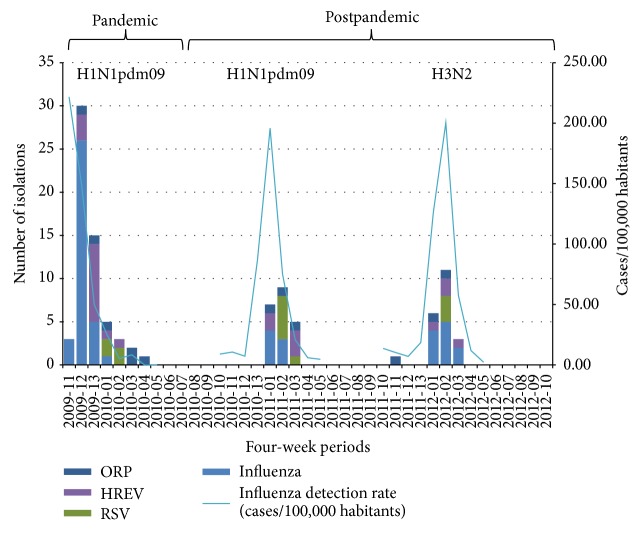
Isolations of respiratory viruses causing AE-COPD episodes and influenza detection rate during the pandemic and the following epidemics included in the study. For the *Y*-axis, the cumulative columns refer to the number of isolations and the lines refer to cases per 100,000 habitants. For the *X*-axis, the timeline represents merged four-week periods beginning with the first week of each year. The influenza viruses most often detected are shown at the top, corresponding with the pandemic and following postpandemic period.

The highest number of AE-COPD episodes (*n* = 124; 63.6%) was recorded for the pandemic, followed by FLUEP1 (*n* = 40; 20.5%) and FLUEP2 (*n* = 25; 12.8%) (Table 2). Only 6 AE-COPD episodes occurred during any of the interepidemic periods, and none of them were diagnosed with respiratory viral infection. The average age of AE-COPD patients during the pandemic, 62.7 ± 13.1 years (Table 2), was not significantly different from the patient ages in the first or second epidemic. The proportion of viral findings in AE-COPD patients increased from the pandemic (*n* = 59; 47.6%) until the end of the study period (Figure 2). The maximum proportion occurred during FLUEP2 (*n* = 16; 64.0%) despite the low number of patients recruited in this period. In contrast, for the general Spanish population, the maximum incidence of influenza occurred during the pandemic, 221.7 cases/100,000 inhabitants, rather than in the following two influenza epidemics, 195.9 cases/100,000 habitants during FLUE1 and 200.3 cases/100,000 habitants during FLUEP2 (Figure 3).

Influenza viruses were the most detected respiratory pathogens in AE-COPD patients during the pandemic and following epidemics. Specifically, influenza A/H1N1pdm09 was detected in 35 cases (28.2%) during the pandemic and in 6 cases (15.0%) during FLUEP1, while H3N2 influenza virus was detected in 11 cases (44.0%) during FLUEP2. Indeed, these two influenza strains represent together 54.7% of viruses diagnosed in AE-COPD episodes in this study. Meanwhile, HREV and RSV were the second and third most diagnosed viruses during the pandemic (*n* = 14; 11.3% and *n* = 4; 3.2%, resp.). However, diagnosis of these two viruses constantly increased in the two following influenza epidemics. Also other respiratory pathogens increased in prevalence in AE-COPD cases from the pandemic (*n* = 6; 4.8%) until FLUEP2 (*n* = 3; 12.0%). Coinfections were more commonly detected during FLUEP2.

We used the OR to analyze the probability of detection of viral categories (ORP, HREV, any influenza virus, and RSV) as well as viral coinfections in AE-COPD patients among different demographic and epidemiological characteristics such as gender, age groups, and the different periods analyzed. There was no gender difference in the rate of detection of respiratory viral or coinfections. The OR for detecting influenza in the 30–64-year age group was 2.78-fold (CI95% = 1.44–5.38) greater than for the ≥65-year old group (*p* = 0.002). The probability for detecting RSV in the pandemic was significantly lower than detecting it in the first epidemic (OR = 0.19; CI95% = 0.05–0.71; *p* = 0.013). Additionally, the probability for detecting influenza virus in the first epidemic was significantly lower than in the second epidemic (OR = 0.27, CI95% = 0.09–0.84; *p* = 0.024). Finally, the probability for detecting coinfection during the pandemic was significantly lower than in the second epidemic (OR = 0.02; IC95% = 0.01–0.37; *p* = 0.009).

## 4. Discussion

Accurate detection of the causes of AE-COPD is important to develop and improve specific therapies and health care for patients that suffer this disease. In this way, clinicians and microbiology laboratories can be better prepared for the constant emergence of new respiratory pathogens such as avian influenza viruses and MERS-coronavirus. Our study has revealed differences between the 2009 pandemic and the following two influenza epidemics and other differences in the etiology dynamic of AE-COPD described in the scientific literature before 2009. Even though most of the AE-COPD respiratory samples were acquired during the pandemic, the viral etiology increased from 47.6% in the pandemic to 64.0% in the FLUEP2. Respiratory viruses affecting AE-COPD episodes have been communicated in epidemics prior to 2009, ranging from 40 to 60% in previous publications [27, 28]. Also in several studies, viruses were associated with higher frequencies of AE-COPD than bacterial infection or air pollution [29]. Furthermore, viral infections serve as causes of secondary bacterial infections that are associated with a rapid decline and severe respiratory symptoms [11, 30, 31]. Our findings are consistent with the global relevance of viruses in AE-COPD as previously described [11, 15, 16]. This suggests that, in addition to the independence of the viral epidemiologic characteristics, there exists a balance between bacterial and viral infections which promotes these exacerbations.

The distribution of AE-COPD patients within the 30–64 and the ≥65-year age groups was similar (48.2 and 51.8%, resp.). Within these age groups, there was a decrease of respiratory viruses in the AE-COPD episodes with increasing age. Thus viral infections were the etiology in 62.2% of AE-COPD episodes in the 30–64-year age group, while accounting for only 38.0% of AE-COPD cases in ≥65-year-old population. These data reveal a clear decrease in the role of respiratory viruses in AE-COPD episodes as the susceptible population ages. Thus, it is likely that bacterial infections or environmental conditions are the most frequent causes of exacerbations in elderly people. Despite the fact that age seems to be a factor for detection of respiratory viruses in AE-COPD episodes, we did not find any differences in the average age between people with respiratory infection and those with negative viral diagnostics. On the other hand, the average age of patients was also similar among the pandemic and influenza epidemics studied.

The virus most frequently detected in AE-COPD patients during the study period was influenza A/H1N1pdm09, followed by HREV and RSV. Analyzing each period, influenza A/H1N1pdm09 was the most frequent pathogen detected in the pandemic and during FLUEP1. RSV was also diagnosed in the same proportion as influenza A/H1N1pdm09 during FLUEP1. On the other hand, influenza A/H3N2 was the most frequently diagnosed pathogen in AE-COPD patients during FLUEP2. Before emergence of the new influenza strain A/H1N1pdm09 in 2009, several works cited HREV as the main etiological agent causing AE-COPD [28, 32]. The prevalence of HREV ranged from 10 to 12% of the total exacerbations, while other viruses like RSV and influenza A and B had prevalences ranging from 6 to 7% and 3 to 4%, respectively [28, 32]. Emergence of this new influenza virus seems to have changed the etiological viral pattern of AE-COPD episodes. It resulted in higher rates of AE-COPD of A influenza viruses compared to other different viral families such as the Picornaviruses at least during the period studied. Despite that, HREV remained as the second most frequent cause of AE-COPD episodes in the patients included in this work.

We analyzed the probability of detection of the different respiratory viruses and coinfections within the demographic and epidemiological characteristics of the study population. Although COPD is a chronic disease that has been historically more associated with males, it has been continuously increasing in women in the recent years [33]. In our study, men represented more than 60% of population with AE-COPD; however, we did not find differences in the detection of any virus or coinfections between men and women.

We also studied the probability of detecting different viruses and coinfections between the two age groups. There were 2.78-fold more influenza virus detections in adults aged 30–64 than in the elderly patients aged ≥65. On the other hand, all of the other viruses were detected at the same proportion in both age groups. Most of the influenza viruses were subtyped as A/H1N1pdm09 influenza strain and were diagnosed during pandemic. This viral strain has strongly affected younger individuals since its emergence in 2009. The apparent tropism of this influenza strain for younger people could be due to a low level or even absence of immunological memory and cross-immunity compared to that present in older individuals, phenomenon well demonstrated as a protective factor in this age group [34].

We also studied the probability of detecting the different respiratory viruses and coinfections among the periods studied in this work. We found differences in detection of RSV in the pandemic and FLUEP1, differences in coinfections in the pandemic and FLUEP2, and differences in influenza detection between the two epidemics. These data support the dynamic etiology of the respiratory viruses on AE-COPD episodes described in this study. Our study shows that the emergence of a new pandemic influenza virus completely changed the etiology of viral infections in AE-COPD patients. These changes were probably caused by the absence of immunity in a large part of the population. This change can be seen in the following years as fluctuations of the viruses causing these exacerbations. For this reason, it is necessary to continue studying this data series to know if the etiology of respiratory viruses can be absolutely changed in AE-COPD by the emergence of a new virus or, alternatively, if this new behavior occurs only for a few years after a pandemic event.

Influenza viruses have been associated with mortality and morbidity in chronic lung disease [31]. Recent studies have focused on the importance of influenza vaccination with emphasis on risk groups such as COPD patients and especially working adults (30–64 years old) with COPD who are not usually covered by vaccination [35, 36]. Also, the early use of antiviral drugs against influenza viruses in hospitalized patient results in better management of AE-COPD episodes [37], especially regarding the high proportion of patients that suffer an infection by influenza viruses causing AE-COPD described in this work. For this reason, it is important to design empiric and rapid laboratory diagnostic strategies to start treatment as soon as possible for these kinds of patients. In connection with that, our data offers highly valuable information based on demographic and epidemiological characteristics that can help clinicians with the diagnosis of AE-COPD patients. Thus the criteria can be adapted to the specific clinical characteristics of each patient and time of the year. Following these data, diagnostic suspicion may be supported on two different aspects: viral infections of AE-COPD patients are more likely in younger patients than in older ones and that detection of viral respiratory infection causing AE-COPD directly depends on influenza epidemic characteristics, at least in the years following a pandemic influenza emergence. Also, these data support the need for multiplex microbiological diagnostic techniques that allow detecting the most frequent viral targets involved in AE-COPD.

The low number of samples after the pandemic limited the performance of more complex statistical analysis. Specifically, the low number of recruited patients during the interepidemic periods impaired the ability to compare epidemics with periods without sustained influenza circulation. The lack of AE-COPD clinical information in some microbiological order slips handled in the laboratory also generated a loss of AE-COPD cases, which could not then be included in the study. However, recognition of this problem has generated a better dynamic on clinical requests completed by clinicians in their medical services. The high percentage of negative samples diagnosed showed the need for improved diagnostics to identify the role of bacterial infections in the AE-COPD in our sanitary area. It is important to continue this study during following influenza epidemics to check for changes in the etiology dynamic after A/H1N1pdm09 has become completely epidemic.

## 5. Conclusions

Emergence of the new influenza pandemic virus A/H1N1pdm09 caused influenza A viruses to be the main pathogens that affected COPD patients during the period studied. However A/H1N1pdm09 did not change the global role of respiratory viruses as the primary cause of COPD exacerbations during the pandemic and following two influenza epidemics. The presence of respiratory viruses in AE-COPD episodes that require hospitalization is related with several demographic and epidemiological factors, such as the age of the patient and characteristics of the influenza epidemic activity. These factors need to be used by clinicians to complete clinical and laboratory diagnostic guides that are focused on the role of the respiratory viruses in exacerbations. Vaccination and antiviral drug use is strongly recommended in these kinds of patients.

## Figures and Tables

**Table 1:** Inclusive study periods.

Period	Description	Duration
PAN	2009 pandemic	Week 35, 2009–week 32, 2010
INEP1	Interepidemic 2010	Week 33, 2010–week 39, 2010
FLUEP1	Influenza epidemic 2010-11	Week 40, 2010–week 20, 2011
INEP2	Interepidemic 2011	Week 21, 2011–week 39, 2011
FLUEP2	Influenza epidemic 2011-12	Week 40, 2011–week 20, 2012
INEP3	Interepidemic 2012	Week 21, 2012–week 39, 2012

**Table 2:** Number and percentage of positives, negatives, gender distribution, average age, and pathogens affecting AE-COPD patients during the entire period and in each separate influenza period included in the study.

	Whole period studied	Pandemic	INEP1	FLUEP1	INEP2	FLUEP2	INEP3
			2010	2010-11	2011	2011-12	2012
AE-COPD cases	195	124	0	40	4	25	2
Mean Age (SD)	63.9 (13.1)	62.7 (13.1)	0 (0)	61.1 (14.2)	66.5 (2.1)	67.9 (12.6)	69.5 (2.1)
Males (%)	136 (69.7)	85 (68.6)	0 (0)	25 (62.5)	2 (50)	22 (88.0)	2 (100)
Negatives (%)	100 (51.3)	65 (52.4)	0 (0)	20 (50)	4 (100)	9 (36.0)	2 (100)
Positives (%)	95 (48.7)	59 (47.6)	0 (0)	20 (50)	0 (0)	16 (64.0)	0 (0)
Pathogen most represented	H1N1pdm09	H1N1pdm09	N/A	H1N1pdm09/RSV	N/A	H3N2	N/A
H1N1pdm09 (%)	41 (21.0)	35 (28.2)	0 (0)	6 (15.0)	0 (0)	0 (0)	0 (0)
H3N2 (%)	11 (5.6)	0 (0)	0 (0)	0 (0)	0 (0)	11 (44.0)	0 (0)
Influenza B (%)	1 (0.5)	0 (0)	0 (0)	1 (2.5)	0 (0)	0 (0)	0 (0)
HREV (%)	24 (12.3)	14 (11.3)	0 (0)	5 (12.5)	0 (0)	5 (20.0)	0 (0)
RSV (%)	13 (6.7)	4 (3.2)	0 (0)	6 (15.0)	0 (0)	3 (12.0)	0 (0)
ORP (%)	12 (6.2)	6 (4.8)	0 (0)	3 (7.5)	0 (0)	3 (12.0)	0 (0)
Coinfections (%)	5 (2.6)	0 (0)	0 (0)	1 (2.5)	0 (0)	4 (16.0)	0 (0)

N/A: data not available; HREV: human rhino/enterovirus; ORP: other respiratory pathogens; I: influenza interepidemic period; E: influenza epidemic period.

## References

[1] MacNee W. (2005). Pathogenesis of chronic obstructive pulmonary disease. *Proceedings of the American Thoracic Society*.

[2] Miravitlles M., Soriano J. B., García-Río F. (2009). Prevalence of COPD in Spain: impact of undiagnosed COPD on quality of life and daily life activities. *Thorax*.

[3] Soriano J. B., Ancochea J., Miravitlles M. (2010). Recent trends in COPD prevalence in Spain: a repeated cross-sectional survey 1997–2007. *European Respiratory Journal*.

[4] Anzueto A., Sethi S., Martinez F. J. (2007). Exacerbations of chronic obstructive pulmonary disease. *Proceedings of the American Thoracic Society*.

[5] Proud D., Chow C.-W. (2006). Role of viral infections in asthma and chronic obstructive pulmonary disease. *American Journal of Respiratory Cell and Molecular Biology*.

[6] Fletcher C., Peto R. (1977). The natural history of chronic airflow obstruction. *British Medical Journal*.

[7] Gómez F. P., Rodriguez-Roisin R. (2002). Global Initiative for Chronic Obstructive Lung Disease (GOLD) guidelines for chronic obstructive pulmonary disease. *Current Opinion in Pulmonary Medicine*.

[8] Gompertz S., Bayley D. L., Hill S. L., Stockley R. A. (2001). Relationship between airway inflammation and the frequency of exacerbations in patients with smoking related COPD. *Thorax*.

[9] Miravitlles M., Guerrero T., Mayordomo C., Sánchez-Agudo L., Nicolau F., Segú J. L. (2000). Factors associated with increased risk of exacerbation and hospital admission in a cohort of ambulatory COPD patients: a multiple logistic regression analysis. The EOLO Study Group. *Respiration*.

[10] Sethi S., Murphy T. F. (2001). Bacterial infection in chronic obstructive pulmonary disease in 2000: a state-of-the-art review. *Clinical Microbiology Reviews*.

[11] Wilkinson T. M. A., Hurst J. R., Perera W. R., Wilks M., Donaldson G. C., Wedzicha J. A. (2006). Effect of interactions between lower airway bacterial and rhinoviral infection in exacerbations of COPD. *Chest*.

[12] Connors A. F., Dawson N. V., Thomas C. (1996). Outcomes following acute exacerbation of severe chronic obstructive lung disease. *The American Journal of Respiratory and Critical Care Medicine*.

[13] Mohan A., Chandra S., Agarwal D. (2010). Prevalence of viral infection detected by PCR and RT-PCR in patients with acute exacerbation of COPD: a systematic review. *Respirology*.

[14] Frickmann H., Jungblut S., Hirche T. O., Groß U., Kuhns M., Zautner A. E. (2012). The influence of virus infections on the course of COPD. *European Journal of Microbiology and Immunology*.

[15] Hutchinson A. F., Ghimire A. K., Thompson M. A. (2007). A community-based, time-matched, case-control study of respiratory viruses and exacerbations of COPD. *Respiratory Medicine*.

[16] McManus T. E., Marley A.-M., Baxter N. (2008). Respiratory viral infection in exacerbations of COPD. *Respiratory Medicine*.

[17] Varkey J. B., Varkey B. (2008). Viral infections in patients with chronic obstructive pulmonary disease. *Current Opinion in Pulmonary Medicine*.

[18] Nseir S., Ader F. (2008). Prevalence and outcome of severe chronic obstructive pulmonary disease exacerbations caused by multidrug-resistant bacteria. *Current Opinion in Pulmonary Medicine*.

[19] Boixeda R., Rabella N., Sauca G. (2012). Microbiological study of patients hospitalized for acute exacerbation of chronic obstructive pulmonary disease (AE-COPD) and the usefulness of analytical and clinical parameters in its identification (VIRAE study). *International Journal of Chronic Obstructive Pulmonary Disease*.

[20] Sullivan S. J., Jacobson R. M., Dowdle W. R., Poland G. A. (2010). 2009 H1N1 influenza. *Mayo Clinic Proceedings*.

[21] Simonsen L., Spreeuwenberg P., Lustig R. (2013). Global mortality estimates for the 2009 influenza pandemic from the GLaMOR project: a modeling study. *PLoS Medicine*.

[22] Paquette S. G., Banner D., Zhao Z. (2012). Interleukin-6 is a potential biomarker for severe pandemic H1N1 influenza a infection. *PLoS ONE*.

[23] Almansa R., Socias L., Andaluz-Ojeda D. (2012). Viral infection is associated with an increased proinflammatory response in chronic obstructive pulmonary disease. *Viral Immunology*.

[24] Anthonisen N. R., Manfreda J., Warren C. P. W. (1987). Antiobiotic therapy in exacerbations of chronic obstructive pulmonary disease. *Annals of Internal Medicine*.

[25] Al Hajjar S., McIntosh K. (2010). The first influenza pandemic of the 21st century. *Annals of Saudi Medicine*.

[26] Biere B., Bauer B., Schweiger B. (2010). Differentiation of influenza b virus lineages yamagata and victoria by real-time PCR. *Journal of Clinical Microbiology*.

[27] Seemungal T., Harper-Owen R., Bhowmik A. (2001). Respiratory viruses, symptoms, and inflammatory markers in acute exacerbations and stable chronic obstructive pulmonary disease. *American Journal of Respiratory and Critical Care Medicine*.

[28] Wedzicha J. A. (2004). Role of viruses in exacerbations of chronic obstructive pulmonary disease. *Proceedings of the American Thoracic Society*.

[29] Bafadhel M., McKenna S., Terry S. (2011). Acute exacerbations of chronic obstructive pulmonary disease: identification of biologic clusters and their biomarkers. *American Journal of Respiratory and Critical Care Medicine*.

[30] Mallia P., Footitt J., Sotero R. (2012). Rhinovirus infection induces degradation of antimicrobial peptides and secondary bacterial infection in chronic obstructive pulmonary disease. *American Journal of Respiratory and Critical Care Medicine*.

[31] Harper S. A., Bradley J. S., Englund J. A. (2009). Seasonal influenza in adults and children-diagnosis, treatment, chemoprophylaxis, and institutional outbreak management: clinical practice guidelines of the Infectious Diseases Society of America. *Clinical Infectious Diseases*.

[32] Greenberg S. B., Allen M., Wilson J., Atmar R. L. (2000). Respiratory viral infections in adults with and without chronic obstructive pulmonary disease. *American Journal of Respiratory and Critical Care Medicine*.

[33] Soriano J. B., Maier W. C., Egger P. (2000). Recent trends in physician diagnosed COPD in women and men in the UK. *Thorax*.

[34] Verma N., Dimitrova M., Carter D. M. (2012). Influenza virus H1N1pdm09 infections in the young and old: evidence of greater antibody diversity and affinity for the hemagglutinin globular head domain (HA1 Domain) in the elderly than in young adults and children. *Journal of Virology*.

[35] Poole P. J., Chacko E., Wood-Baker R. W., Cates C. J. (2000). Influenza vaccine for patients with chronic obstructive pulmonary disease. *The Cochrane Database of Systematic Reviews*.

[36] Nichol K. L., Nordin J. D., Nelson D. B., Mullooly J. P., Hak E. (2007). Effectiveness of influenza vaccine in the community-dwelling elderly. *The New England Journal of Medicine*.

[37] Kaiser L., Wat C., Mills T., Mahoney P., Ward P., Hayden F. (2003). Impact of oseltamivir treatment on influenza-related lower respiratory tract complications and hospitalizations. *Archives of Internal Medicine*.

